# Supplementary material for: Mechanisms governing the pioneering and redistribution capabilities of the non-classical pioneer PU.1
Source: Nat Commun. 2020 Jan 21;11:402. doi: 10.1038/s41467-019-13960-2 (PMC6972792; doi:10.1038/s41467-019-13960-2)
Supplement: Supplementary file 7 — Source data [file 41467_2019_13960_MOESM7_ESM.zip › Source_Data/FigureS2/FigureS2B_MotifScanOutput/KmeansCluster14/homerResults/motif12.similar.html]

motif12

## Information for motif12

T
A
G
C
C
G
T
A
T
A
C
G
C
G
T
A
A
C
G
T
G
T
C
A
C
G
T
A
A
C
T
G
T
G
C
A
  
Reverse Opposite:  

A
C
G
T
G
T
A
C
A
C
G
T
C
A
G
T
C
G
T
A
C
G
A
T
A
T
G
C
C
G
A
T
A
T
C
G
  

|  |  |
| --- | --- |
| p-value: | 1e-18 |
| log p-value: | -4.338e+01 |
| Information Content per bp: | 1.833 |
| Number of Target Sequences with motif | 47.0 |
| Percentage of Target Sequences with motif | 9.22% |
| Number of Background Sequences with motif | 788.2 |
| Percentage of Background Sequences with motif | 1.74% |
| Average Position of motif in Targets | 103.5 +/- 52.1bp |
| Average Position of motif in Background | 98.1 +/- 63.8bp |
| Strand Bias (log2 ratio + to - strand density) | 0.5 |
| Multiplicity (# of sites on avg that occur together) | 1.09 |
| Motif File: | file (matrix) reverse opposite |

### Similar de novo motifs found

|  |  |  |  |  |  |  |  |
| --- | --- | --- | --- | --- | --- | --- | --- |
| Rank | Match Score | Redundant Motif | P-value | log P-value | % of Targets | % of Background | Motif file |
| 1 | 0.931 | G T A C C G A T T A G C C G T A C T A G C G T A C G A T G T C A C G T A A C T G T C G A | 1e-16 | -37.153252 | 4.51% | 0.39% | motif file (matrix) |
| 2 | 0.853 | A T G C A C G T A C G T C G T A A C G T A T G C C G T A | 1e-14 | -33.544496 | 10.98% | 3.16% | motif file (matrix) |
| 3 | 0.897 | C G T A T C A G G T C A A C G T C G T A C G T A C A T G G T A C G A T C G T A C | 1e-13 | -30.881068 | 4.90% | 0.65% | motif file (matrix) |
| 4 | 0.946 | A G T C C G T A A C T G C G T A A C G T G T C A C G T A T C A G | 1e-13 | -29.992182 | 5.69% | 0.95% | motif file (matrix) |
| 5 | 0.942 | C G T A A C T G C G T A A C G T C G T A C G T A A C T G G T C A | 1e-12 | -28.759184 | 6.27% | 1.24% | motif file (matrix) |
